# Epidemiological Features of COVID-19 in Northwest Russia in 2021

**DOI:** 10.3390/v14050931

**Published:** 2022-04-29

**Authors:** Anna Gladkikh, Vladimir Dedkov, Alena Sharova, Ekaterina Klyuchnikova, Valeriya Sbarzaglia, Olga Kanaeva, Tatyana Arbuzova, Nadezhda Tsyganova, Anna Popova, Edward Ramsay, Areg Totolian

**Affiliations:** 1 Saint Petersburg Pasteur Institute, 14 Ulitsa Mira, 197101 Saint Petersburg, Russia; angladkikh@gmail.com (A.G.); alenasharova21@gmail.com (A.S.); erithrophtalmis@gmail.com (E.K.); sbarzaglia.valeriya@gmail.com (V.S.); ol.kanaeva@gmail.com (O.K.); arbuzowa95@yandex.ru (T.A.); lukyanchuk_np@pasteurorg.ru (N.T.); warmsunnyday@mail.ru (E.R.); totolian@spbraaci.ru (A.T.); 2 Martsinovsky Institute of Medical Parasitology, Tropical and Vector Borne Diseases, Sechenov First Moscow State Medical University, 119435 Moscow, Russia; 3 Federal Service on Consumer Protection and Human Well-Being Surveillance, 127994, Moscow, Russia; depart@gsen.ru

**Keywords:** SARS-CoV-2, COVID-19, variants of concern, AT.1 variant, Russia

## Abstract

Appearing in Wuhan (China) and quickly spreading across the globe, the novel coronavirus infection quickly became a significant threat to global health. The year 2021 was characterized by both increases and decreases in COVID-19 incidence, and Russia was no exception. In this work, we describe regional features in the Northwestern federal district (FD) of Russia of the pandemic in 2021 based on Rospotrebnadzor statistics and data from SARS-CoV-2 genetic monitoring provided by the Saint Petersburg Pasteur Institute as a part of epidemiological surveillance. The epidemiological situation in the studied region was complicated by the presence of the megacity Saint Petersburg, featuring a high population density and its status as an international transport hub. COVID-19 incidence in the Northwestern FD fluctuated throughout the year, with two characteristic maxima in January and November. An analysis of fluctuations in the age structure, severity of morbidity, mortality rates, and the level of population vaccination in the region during the year is given. Assessment of epidemiological indicators was carried out in relation to changes in locally circulating genetic variants. It was seen that, during 2021, so-called variants of concern (VOC) circulated in the region (Alpha, Beta, Delta, Omicron), with Delta variant strains dominating from June to December. They successively replaced the variants of lines 20A and 20B circulating at the beginning of the year. An epidemiological feature of the northwestern region is the AT.1 variant, which was identified for the first time and later spread throughout the region and beyond its borders. Its share of the regional viral population reached 28.2% in May, and sporadic cases were observed until September. It has been shown that genetic variants of AT.1 lineages distributed in Russia and Northern Europe represent a single phylogenetic group at the base of the 20B branch on the global phylogenetic tree of SARS-CoV-2 strains. The progression of the COVID-19 pandemic occurred against the background of a vaccination campaign. The findings highlight the impact of vaccination on lowering severe COVID-19 case numbers and the mortality rate, despite ongoing changes in circulating SARS-CoV-2 genetic variants.

## 1. Introduction

More than two years have passed since the beginning of the COVID-19 pandemic, occurring in late December 2019 in Wuhan, Hubei Province (China). When SARS-CoV-2 was identified as the causative agent of COVID-19, no one expected that the outbreak at the Huanan Seafood Market would grow into such a prolonged global pandemic. The origins of SARS-CoV-2 remain unknown, as does the exact date of the initial outbreak [1]. As of 6 April 2022, 492,189,439 COVID-19 cases have been identified, with 6,159,474 fatalities [2]. Despite a number of successes in the fight against the pandemic, including the creation of vaccines, specific antiviral drugs, and the optimization of treatment/prevention regimens, there is no hope for an end to the pandemic this year. Russia, as part of the global community, was also affected by the pandemic. The first COVID-19 patient in Russia was registered on 2 March 2020 [3]. As of 6 April 2022, 17,940,765 COVID-19 cases have been identified in Russia, with 370,602 fatalities [4].

Regional differences in the epidemic process occur due to Russia’s expansive geography and the uneven population of its different federal districts (FDs). In two of them, the Central FD and Northwestern FD, the majority of new COVID-19 cases are registered. In addition, the spread of emerging SARS-CoV-2 genetic variants usually begins in these regions. This is due to their high population densities, the presence of two megacities (Moscow in the Central FD and Saint Petersburg in the Northwestern FD), and to the fact that megacities are the largest transport hubs carrying the most international traffic.

Here, we present data characterizing the COVID-19 epidemic in Russia’s northwestern region (total population 13,941,919) in 2021. The epidemiological features of COVID-19 in this region are all the more interesting because a variant under monitoring (VUM), AT.1, was initially identified here [3]. In our study, we used statistical data from Rospotrebnadzor as well as data from the genetic monitoring of SARS-CoV-2 implemented by the Saint Petersburg Pasteur Institute.

## 2. Materials and Methods

### 2.1. Epidemiological Data

In our study, we used statistical data from Rospotrebnadzor, obtained from an internal database based on daily regional reports.

### 2.2. Study Samples

During routine study of SARS-CoV-2 genetic diversity in Russia from January 2021 until January 2022, 5032 nasopharyngeal swabs from COVID-19 patients, admitted to hospitals or health centers located in different regions of northwest Russia, were collected and delivered to the Saint Petersburg Pasteur Institute for sequencing and further phylogenetic study (Table 1). In addition, 3317 swabs were studied using RT-PCR for the estimation of AT.1 prevalence in Saint Petersburg. The swabs were collected in 500 µL of special transport medium or phosphate-buffered saline (pH 7.0) and stored at −20 °C until analysis.

### 2.3. RNA Extraction and Reverse Transcription qPCR

The total nucleic acid samples were obtained by extraction and purification using the RIBO-prep DNA/RNA Extraction Kit (AmpliSens^®^, Moscow, Russia) according to the manufacturer recommendations. DNA/RNA was eluted with 50 µL of elution buffer and stored at −70 °C until molecular analysis. For SARS-CoV-2 detection and to assess viral load, the nucleic acids from swabs were thoroughly analyzed using the COVID-19 Amp RT-qPCR Kit (Saint Petersburg Pasteur Institute, St. Petersburg, Russia) according to the manufacturer recommendations [5]. SARS-CoV-2 positive samples, featuring Ct values of 25 or less, were selected and studied further using high throughput or Sanger sequencing.

### 2.4. Library Preparation and Near-Complete Genome Sequencing

Reverse transcription was performed using random hexanucleotide primers and the Reverta-L Kit (AmpliSens^®^, Moscow, Russia) according to the manufacturer instructions; cDNA samples were stored at −70 °C and subsequently used as amplification templates. Libraries were prepared using an in-house primer panel and sequenced using the Illumina MiSeq System (Illumina Inc., San Diego, CA, USA) with the MiSeq Reagent Kit v3 (600 cycle) (Illumina Inc., San Diego, CA, USA), as described earlier [6].

### 2.5. Genome Assembly

The quality of Illumina reads was assessed using the FastQC program [7]. Raw reads were filtered with Trimmomatic [8] to remove adapters, low-quality nucleotides, and biased sequences at the ends of reads (parameters ILLUMINACLIP:TruSeq3-PE.fa:2:30:10:2 SLIDINGWINDOW:4:20 LEADING:3 TRAILING:3 MINLEN:50). Genome assembly was carried out by mapping to the SARS-CoV-2 reference genome (Wuhan-Hu-1 strain, NCBI accession number NC_045512.2) using samtools and bcftools software [9]. The Nextclade tool was used to assess the quality of assembled sequences and to assign genomes into lineages [10]. Sequences were uploaded to GISAID under the following IDs: EPI_ISL_11225838; EPI_ISL_11246080; EPI_ISL_11246081; and EPI_ISL_11267880–EPI_ISL_11267908.

### 2.6. Phylogenetic Reconstruction

The alignment of nucleotide sequences was performed in MAFFT v. 7.475 [11]. In addition to the sequences obtained in this study, sequences from the GISAID database were used. For the phylogeny of AT.1 strains, a substitution model estimation and ML phylogeny was constructed with MEGA X software [12]. A global phylogenetic tree of SARS-CoV-2 variants was constructed using the tools implemented in Nextclade [10].

### 2.7. RT-qPCR Assay for Detection of the AT.1 SARS-CoV-2 Genetic Variant

An assay targeting the 23598-23599 insertion (S gene) of the AT.1 SARS-CoV-2 genetic variant was developed for the estimation of AT.1 prevalence in Saint Petersburg. The sample reaction volumes were 25 µL total, containing the following: 1 µL of BioMaster Mix (Biolabmix, Novosibirsk, Russia); 12.5 µL of 2× reaction buffer (Biolabmix, Novosibirsk, Russia); 0.25 µL of each primer and probe (NWf, tgcgctagttatcagactcag; NWr, tggcaatagagttattagagtaag; NWpr, R6G-cccagcagttaaataacagcatgatcatgtg-BHQ1) with final concentrations of 0.4 µM for primers and 0.28 µM for probes; and 10 µL of the RNA sample. The amplification program was: 50 °C for 15 min; 95 °C for 5 min; followed by 40 cycles (95 °C for 10 s and 57 °C for 30 s). Reactions were performed using the CFX96 thermal cycler (Bio-Rad, Hercules, CA, USA). Fluorescence was registered at the 57 °C step using HEX. The fluorescence threshold was established from the positive-control sample as: the middle value of the linear rise in fluorescence (logarithmic graph). Amplification results were considered positive if the fluorescence level crossed the threshold. As RT-PCR controls, an external positive control for PCR (C^+^) and an armored recombinant positive control for reverse transcription (ARC^+^) were applied [5]. In addition, negative controls for extraction (EC^−^) and PCR (C^−^) were used to exclude false-positive results due to potential unintentional cross-contamination.

## 3. Results

**Figure 1:**
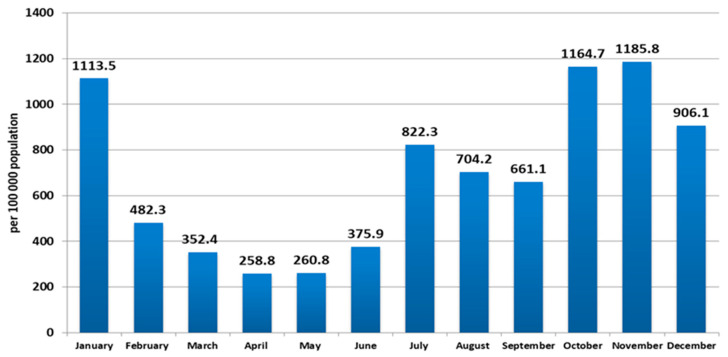
COVID-19 incidence in northwest Russia in 2021. Periodic increases and decreases in incidence can be noted. Figure based on Rospotrebnadzor data.

In 2021, COVID-19 incidence in northwest Russia varied in waves, ranging from 258.8 (per 100 K/month) in April to 1185.8 in November. At the same time, increases in incidence with the achievement of local maxima were recorded in January (1113.5 per 100 K/month), June (822.3 per 100 K/month), and November (1185.8 per 100 K/month) (Figure 1).

**Figure 2:**
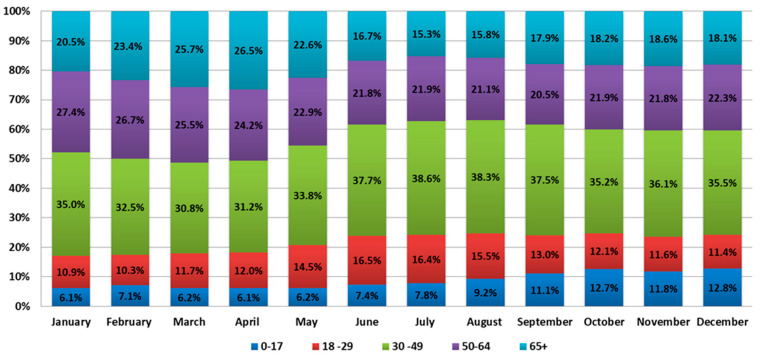
Changes in the demographic structure of COVID-19 incidence in 2021. The proportion of children among new COVID-19 cases increased twofold in 2021. Figure based on Rospotrebnadzor data.

An analysis of the age structure of COVID-19 incidence showed the greatest fluctuations in the age group from 0 to 17 years. In January, COVID-19 incidence in this group was 6.1% of cases, while in December, the incidence was 12.8% of cases. During 2021, there was a gradual increase in incidence in this age group. In the other age groups at the same time, the fluctuations were not so significant and were as follows: from 10.9% to 16.5% in the age group 18–29 years; from 30.8% to 38.6% in the age group 30–49 years; from 20.5% to 27.4% in the age group 50–64 years; and from 15.3% to 26.5% in the group 65^+^ year (Figure 2).

**Figure 3:**
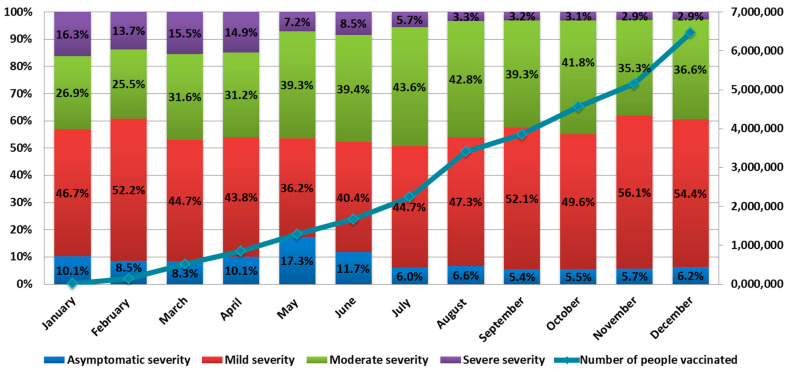
COVID-19 clinical severity and the number of vaccinated individuals in 2021. Concurrent with a substantial rise in the number of vaccinated individuals, the number of severe cases decreased by more than fivefold. Figure based on Rospotrebnadzor data.

The number of cases of severe COVID-19 in 2021 ranged from 16.3% in January to 2.9% in December. The number of cases of asymptomatic infection ranged from 5.4% in September to 17.3% in May. Mild forms ranged from 36.2% in May to 56.1% in November. The number of COVID-19 cases with moderate symptoms ranged from 26.9% in January to 43.6% in July (Figure 3).

**Figure 4:**
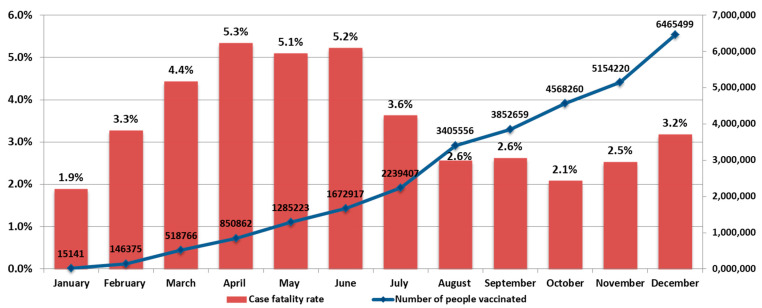
COVID-19 mortality rate and the number of vaccinated individuals in 2021. Concurrent with a substantial rise in the number of vaccinated individuals, the number of deaths decreased by 1.7-fold. Figure based on Rospotrebnadzor data.

The COVID-19 mortality rate also varied in waves, ranging from 1.9% in January to 5.3% in April. Local maxima in mortality were seen in April (5.3%), September (2.6%), and December (3.2%) (Figure 4).

**Figure 5:**
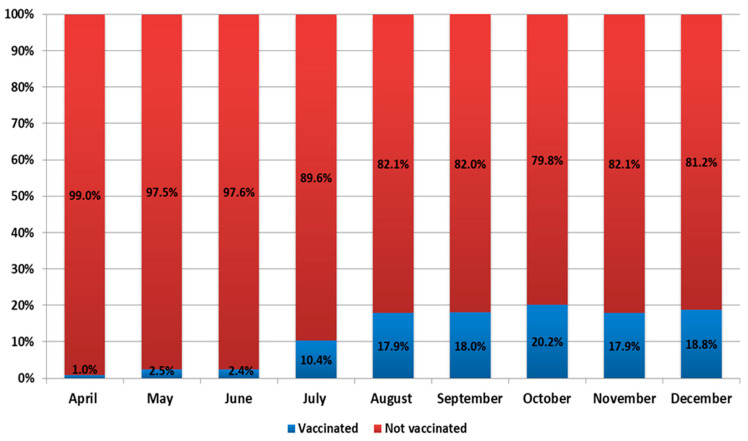
Share of vaccinated persons among SARS-CoV-2 positive samples. The increase in the share of vaccinated persons among newly identified COVID-19 cases confirms that vaccination does not provide 100% protection against SARS-CoV-2 infection. Figure based on Rospotrebnadzor data.

It should be noted that, during 2021, events developed against the background of a vaccination campaign. The number of vaccinated individuals increased from 15,141 in January to 6,465,499 in December (Figure 4 and Figure 5). In 2021, there was an increase in the share of samples from vaccinated individuals in the total volume of positive samples detected. For instance, the share of vaccinated persons among positive samples ranged from 1% in January to 18.8% in December (Figure 5).

**Figure 6:**
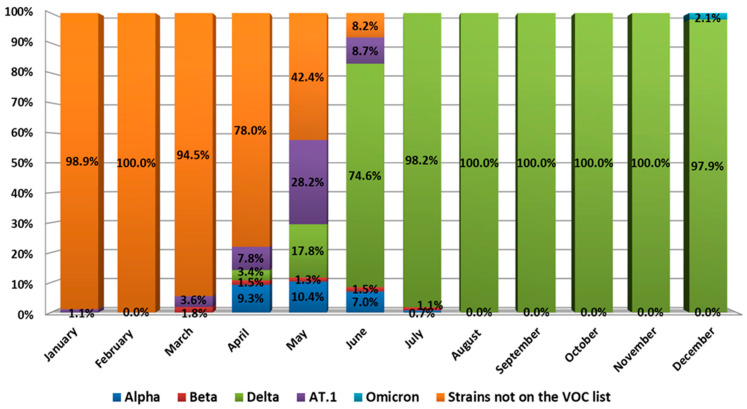
SARS-CoV-2 genetic diversity in northwest Russia. From January to May 2021, various SARS-CoV-2 genetic variants, including VOCs, circulated in northwest Russia. However, from the beginning of June, they were completely displaced by the Delta variant.

SARS-CoV-2 genetic diversity in northwest Russia also varied and was characterized by the presence of variants of concern (VOC) (Figure 6).

During the first quarter of 2021, the 20B lineage SARS-CoV-2 variant dominated in northwest Russia. However, along with wild type, we detected the Beta SARS-CoV-2 variant, which is one of the so-called variants of concern (VOCs) [13]. In addition, we detected the AT.1 SARS-CoV-2 variant in January 2021, which was admitted as a variant under monitoring (VUM) [3]. Its prevalence in January reached 1.1%. It stayed low during the first ten days of 2021 but reached 3.6% in March.

The genetic landscape of SARS-CoV-2 changed in April. The share of wild type genetic variants dropped to 78%. At the same time, circulation of the Alpha and Delta variants of concern began, with Alpha reaching 9.3%. The share of Beta variants remained low as Delta variants appeared in the population. AT.1 prevalence also reached 7.8%.

A significant change in the genetic landscape began in May 2021. Displacement of the wild-type virus continued, and its prevalence decreased to 42.4%. At the same time, AT.1 prevalence reached 28.2%, while Delta variant prevalence was 17.8%. The Alpha variant constituted 10.4% of all genetic variants in May. Such Delta variant dynamics led to the fact that, by the end of June, there was almost a complete displacement of all other SARS-CoV-2 variants. Thus, Delta variant prevalence in July was 97.1%, while wild type (20B) prevalence was only 2.9%.

**Figure 7:**
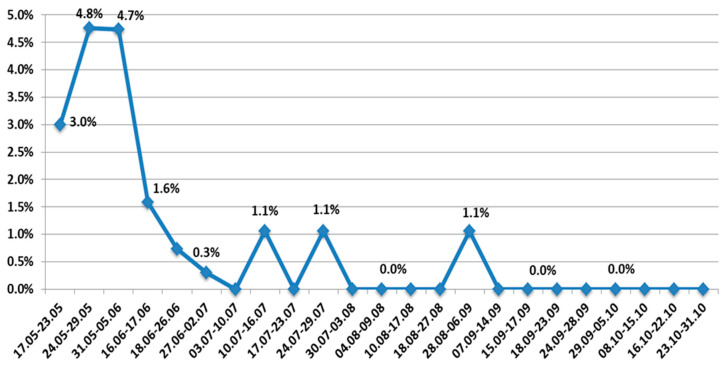
Share of AT.1 among SARS-CoV-2 genetic variants in northwest Russia in 2021.

From the beginning of July to the middle of December, a complete dominance of the Delta variant was noted. Other VOCs, as well as wild type SARS-CoV-2, were eliminated. At the same time, the AT.1 SARS-CoV-2 variant continued to circulate sporadically until early September 2021 (Figure 7).

The variant ceased to circulate widely with the advent of the Delta variant, although AT.1 continued to be sporadically detected until September 2021. In mid-December, an Omicron SARS-CoV-2 variant was identified in northwest Russia. By the end of 2021, Omicron prevalence was 2.1%, while Delta prevalence was 97.9%.

### 3.1. SARS-CoV-2 Genetic Diversity in Northwest Russia

**Figure 8:**
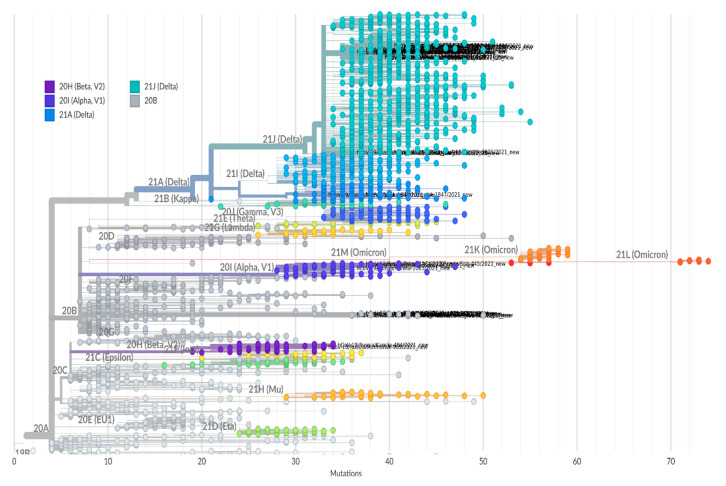
SARS-CoV-2 phylogenetic tree reconstruction based on Nextclade tools. Branches are labeled and colored by clade according Nextstrain nomenclature (legend top left). Sequences obtained in this study are highlighted with their names in black. The scale bar at the bottom indicates the number of nucleotide differences between each sample and the Wuhan-Hu-1/2019 reference sequence (GenBank: MN908947).

Genomic analysis based on Nextclade SARS-CoV-2 Clade Assigner showed seven lineages (Figure 8) circulating in the region in 2021: 20A, 20B, 20H (Beta); 20I (Alpha); 21A (Delta); 21J (Delta); and 21K (Omicron). Since July, the majority of isolates belonged to 21J (Delta).

### 3.2. Phylogeny of the AT.1 Lineage

Phylogenetic analysis of AT.1 strains was performed with all of the sequences available in the GISAID database. According to the Pangoline database [14], AT.1 isolates were identified in Russia, Finland, Estonia, Lithuania, Bulgaria, Austria, Germany, and Belgium. The AT.1 lineage does not form a separate cluster on the global tree of SARS-CoV-2 strains (Figure 8) but forms a long lineage within clade 20B.

The AT.1 genetic variant is characterized by the presence of five non-synonymous substitutions (P9L, D215G, H245P, E484K, E780K) as well as a long deletion (136–144) and an insertion (679) in the S gene. Analysis of 150 AT.1 sequences from GISAID has shown that not all of them share these mutations or characteristic insertion and deletion. For this reason, only sequences with characteristic insertion and deletion (127 sequences) were chosen for phylogenetic analysis. In total, 25 variable sites were identified, 7 of which were non-singleton. Translated S gene sequence had 16 substitutions, 3 of which were non-singleton. Mutations D215G and H245P were not universal: 5.5% of sequences contained D at position 215 like the reference genome S gene; and 3.9% of sequences contained H at position 245 like the reference genome. Position 614 featured G or D variability.

**Figure 9:**
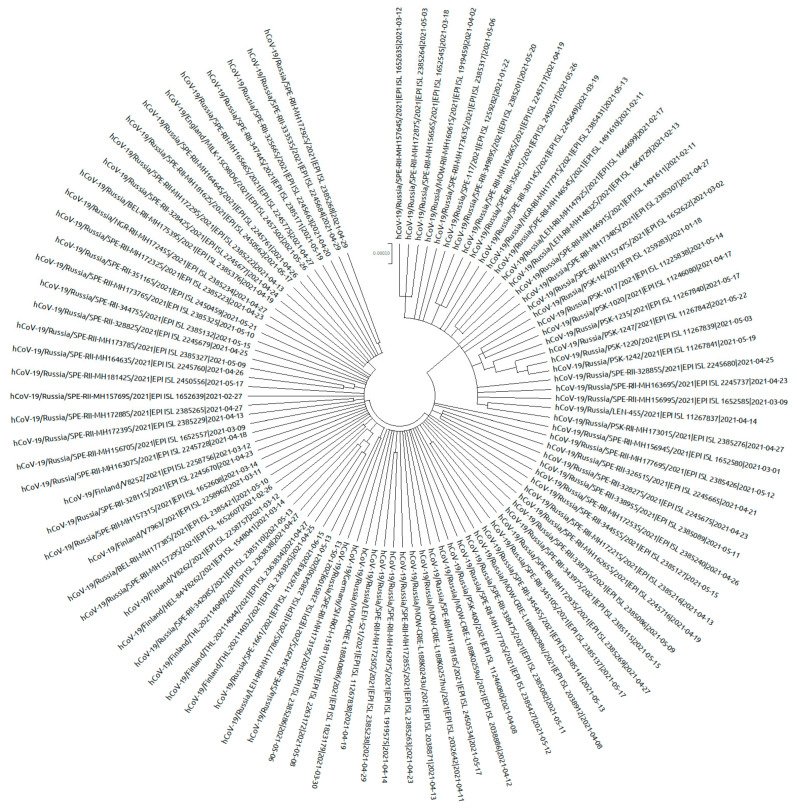
Phylogenetic tree of AT.1 SARS-CoV-2 variants. Sequences (98) from Russia and Europe were included in the analysis. The TN93 substitution model was chosen as optimal for 19,205 bp without gaps/missing data alignment.

**Figure 10:**
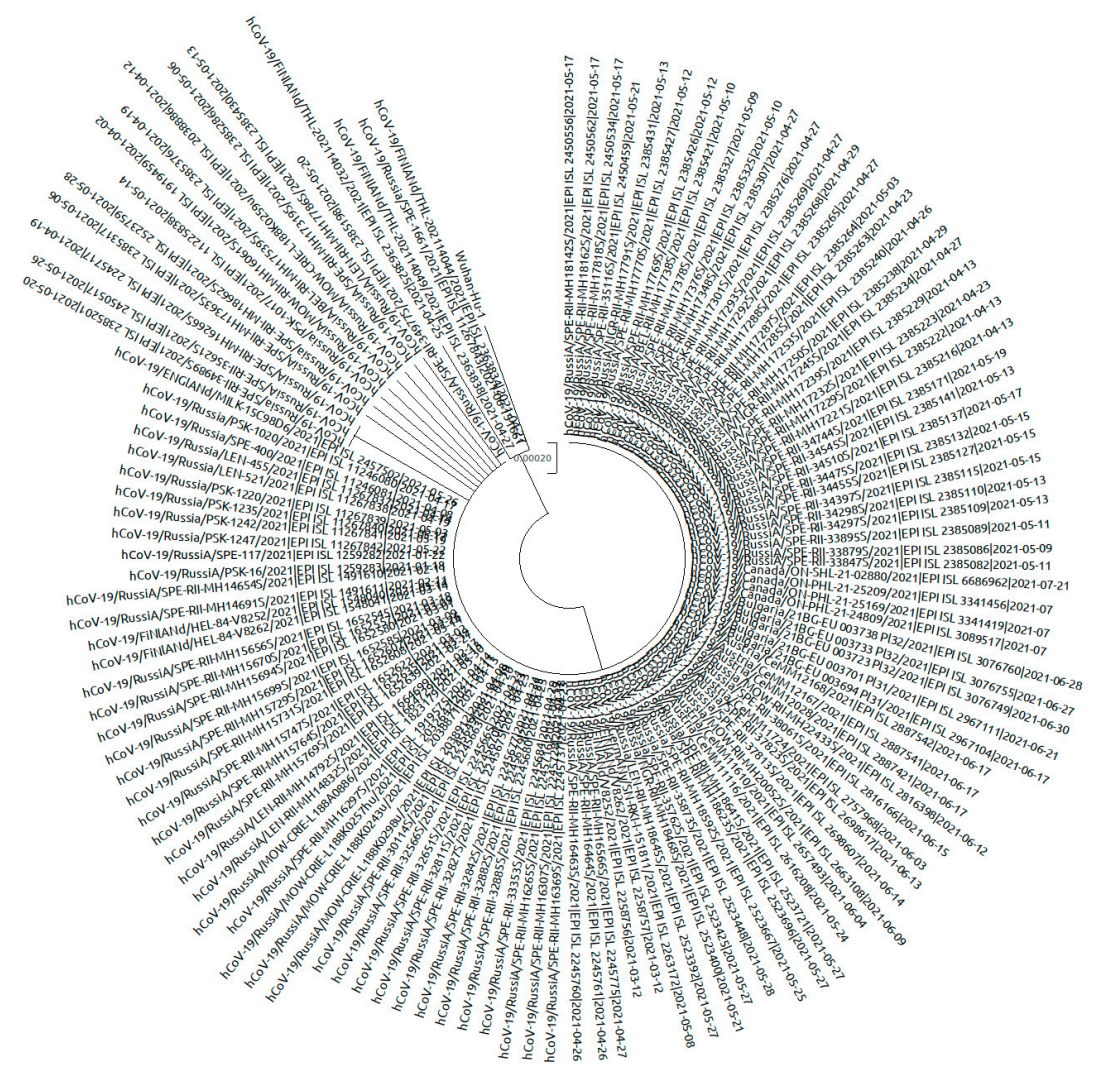
Phylogenetic tree of AT.1 SARS-CoV-2 variants based on S gene sequence alignment. Sequences (127) from Russia and Europe were included in the analysis. The TN92 substitution model was chosen as optimal. Wuhan-Hu-1 sequence used as outgroup (GenBank: NC_045512).

The phylogenetic tree was constructed with sequences from Russia, Finland, Germany, and England. Intra-lineage diversity represented: up to 48 nucleotides in difference between each pair of sequences per genome alignment; and up to 5 nucleotides in difference between each pair of sequences within S gene alignment. On the phylogenetic tree, sequences from northwest Russia (Saint Petersburg, Leningrad region, Novgorod region, Pskov region) were mixed together with sequences from other regions (Moscow, Belgorod region, Yaroslavl region) and sequences from outside of Russia (Figure 9 and Figure 10).

### 3.3. Variant of Concern Genetic Diversity in the Northwest Region

For 20I (Alpha) variants, 254 sequences from Russia were analyzed in total. Pairwise distance was up to 26 nucleotides per genome and up to 6 nucleotides for the S gene. Within S gene sequences from Russia, 114 variable sites were identified, of which 29 were non-singletons. For amino acid alignment, 62 variable sites were identified, of which 16 were non-singletons. For sequences from northwest Russia (75 sequences), 34 nucleotide sites were variable, 8 of which were non-singletons. Regarding amino acid sequence, there were 18 amino acid substitutions 5 of which were singletons. Non-singleton substitutions were at S gene positions 67 (A/V), 502 (Y/N), 571 (A/D), 615 (G/D), and 846 (A/S).

On the global phylogenetic tree, two sequences from northwest Russia obtained within this work (CoV-19/Russia/SPE-445/2021, hCoV-19/Russia/PSK-1464/2021) were clustered with sequences from Germany (Germany/NW-RKI-I-093558/2021). Sequence hCoV-19/Russia/KR-1063/2021 clustered with sequences from England and Denmark. Sequence hCoV-19/Russia/MUR-466/2021 clustered with mixed sequences from Europe, and sequence hCoV-19/Russia/VLG-1249/2021 was located more closely to an isolate from England (England/PORT-2E5324/2021).

For 20H (Beta) variants, 33 sequences from Russia were analyzed. Pairwise distance was up to 22 substitutions per genome and up to 6 substitutions in the S gene. Within S gene sequences from Russia, 16 variable sites were identified, 4 of which were non-singletons. For amino acid alignment, 10 variable sites were seen, of which 3 were non-singletons. For sequences from northwest Russia (8 sequences), 4 nucleotide sites were variable, 2 of which were non-singletons. Regarding amino acid sequences, there were 2 amino acid substitutions, one of which was singleton. The non-singleton substitution was at position 18 (F/L) in the S gene.

On the global phylogenetic tree, one sequence from northwest Russia obtained within this work (hCoV-19/Russia/MUR-969/2021) was located in the cluster with sequences from the USA, South Africa, and Germany. Another (hCoV-19/Russia/KR-494/2021) was located in the cluster with sequences from Wales and England.

The first Delta strains began to appear in the northwest region in April, belonging to two clades: 21A and 21J. The within-genome pairwise difference for 21A was 11 nucleotides and up to 2 nucleotides for the S gene. For sequences from northwest Russia (6 sequences), 21 nucleotide sites were variable within the S gene, 1 of which was non-singleton. Regarding the amino acid sequence, there were 2 amino acid substitutions, 1 of which was non-singleton. The non-singleton substitution was at position 950 (N/D) of the S gene.

For 21J, pairwise differences were up to 15 nucleotides for the complete genome and up to 4 nucleotides for the S gene. For sequences from northwest Russia (56 sequences), 23 nucleotide sites were variable within the S gene, 3 of which were non-singletons. Regarding amino acid sequences, there were 20 amino acid substitutions, 3 of which were singletons. Non-singleton substitutions were at S gene positions 19 (T/R), 614 (D/G), and 950 (N/D).

Sequences from clade 21A were closely related to the Canada/MB-NML-86772/2021 sequence. Sequences from 21J were more abundant. On the SARS-CoV-2 global tree, they formed two groups: one located in a basal position of 21J (closest relative England/NORT-1BD1AFD/2021); and the other forming a subcluster (closest relative Finland/THL-202122712/2021) on the tree.

## 4. Discussion

Systematic monitoring of key epidemiological indicators is an integral part of anti-epidemic measures, both at the regional and national levels. The modern development of molecular technologies has made it possible to study the genetic variability of SARS-CoV-2 and link it with epidemiological indicators since the beginning of the COVID-19 pandemic. All viruses, including SARS-CoV-2, change their genetic structure during circulation. Most events associated with a change in genetic sequence do not lead to changes in the biological properties of the pathogen. However, some mutations can cause changes in a number of properties, such as infectious activity, pathogenicity, the ability to escape post-vaccination immunity, etc.

As a result of intense and widespread circulation, SARS-CoV-2 genetic variants, which pose an increased risk to global health, began to appear in the second half of 2020. This circumstance prompted the WHO to introduce warning classifications for such SARS-CoV-2 genetic variants. It is in this context that the VOC, VOI, and VUM definitions appeared. Such classification made it possible to create guidelines for evaluating SARS-CoV-2 genetic monitoring data. It also contributed to the adoption of optimal management solutions related to anti-epidemic measures.

The appearance of new genetic variants is a natural result of SARS-CoV-2 evolution. At the same time, some variants, being more aggressive, are able to displace others. Such features were first noted with the British genetic variant, which appeared in England in the autumn of 2020. From the beginning of 2021, it began to rapidly spread around the world and displace genetic variants circulating at that time [15]. Being the first VOC, the British (Alpha) genetic variant was defined by the presence of 23 nucleotide mutations across the genome that map to a single branch of the phylogenetic tree [16]. The presence of a wide range of mutations, including in the RBD (the site responsible for cellular ACE2 receptor binding), contributed to its increased infectious activity and a change in the clinical manifestation of COVID-19 [15,17]. The Alpha SARS-CoV-2 variant was obtained in northwest Russian at the end of 2020. This VOC spread throughout the region and accounted for up to 10% of sequenced samples by May 2021.

The first variant capable of totally displacing others was the Delta genetic variant. It was first discovered in October 2020 in India and demonstrated the highest transmissibility rate seen during the study period. Despite the fact that the previous variants of concern (Alpha, Beta, Gamma) had spike protein mutations, the presence of a special mutation signature (L452R, T478K, P681R) in the S protein made it very highly transmissible. The transmissivity of this strain is 97% higher than that of the Wuhan variant [18]. By September 2021, the dominance of the Delta genetic variant was widespread. In the northwestern region, the Delta variant totally displaced the others by July 2021 and totally dominated for six months. The Omicron genetic variant was detected in the northwestern region in December 2021 and is rapidly spreading around the world at the present time.

Other variants are less aggressive than VOCs, but their occurrence indicates local signs of viral evolution. Hence, after appearing in the northwestern region, a variant belonging to the AT.1 lineage reached 28.2% of the viral population in the region and actively competed with other genetic variants circulating at that time. Only the appearance of the Delta genetic variant, which totally displaced all others, reduced AT.1 and led to elimination in the region, although sporadic cases were noted until September 2021.

This paper presents a comprehensive analysis of the COVID-19 situation in northwest Russia in 2021, including not only epidemiological indicators but also data on genetic variants. Epidemiological data were taken from a non-public Rospotrebnadzor database that is updated daily, which allows for the objective assessment of the epidemiological situation at specific time points. The genetic structure of the viral population, on the contrary, cannot be studied in its entirety due to the limited selection of strains suitable for sequencing. This, to some degree, limits the identification of rare variants in the viral population. The appearance of the AT.1 variant in the region makes clear that unnoticed sporadic appearance or disappearance of other rare genetic variants cannot be ruled out.

The epidemic process in northwest Russia was characterized by periodic rises and falls in incidence. These could be associated with, among other things, the predominance of certain SARS-CoV-2 genetic variants. The epidemic course continued alongside increasing vaccination, which has been actively conducted since December 2020, when vaccines were developed in Russia [19,20,21]. By the end of 2021, 45.7% of the Russian population (66,540,512 people) were vaccinated. At the same time, 46.4% of the population (6,465,494 people) were vaccinated in Russia’s Northwestern Federal District [22]. It should be noted that the majority of people were vaccinated with *Gam-COVID-Vac*, better known as Sputnik V [19]. In this context, there were decreases in the mortality rate and in the severity of COVID-19 clinical manifestation. Thus, while vaccination could not completely stop the epidemic process, it nevertheless had a positive effect on it. This effect persists, to one degree or another, despite a decrease in the strength of post-vaccination immunity against newly emerging SARS-CoV-2 variants. The reduction in the risk of SARS-CoV-2 infection in vaccinated persons is indirectly indicated by the fact that, during 2021, the share of children among COVID-19 cases (who did not participate in the immunization program) increased by twofold.

## 5. Conclusions

Here, we have described the epidemiological features of COVID-19 and the genetic variability of SARS-CoV-2 in northwest Russia in 2021. The COVID-19 pandemic in 2021 was characterized by a wave-like course and changes in SARS-CoV-2 genetic variants, including the increasing appearance of emerging genetic variants. During the pandemic, all countries took all possible measures to reduce COVID-19 incidence, including vaccination.

In Russia, there is a high level of vaccine availability. However, the overall vaccination rate is average, which is mainly due to low population compliance with the vaccination program. In this sense, northwest Russia differs little from other regions in the country. Nevertheless, the vaccination campaign has reduced the number of severe COVID-19 cases, while decreasing the mortality rate, despite ongoing changes in circulating SARS-CoV-2 genetic variants.

## Figures and Tables

**Table 1:** Number and origin of sequences used in the study.

No.	Region	Number of Sequences
1	Karelia Republic	329
2	Komi Republic	289
3	Arkhangelsk region	351
4	Vologda region	1028
5	Kaliningrad region	369
6	Leningrad Region	331
7	Murmansk region	497
8	Novgorod region	73
9	Pskov region	907
10	Saint Petersburg	846
11	Nenets Autonomous District	12
**Total**		**5032**
