# Correction: Gladkikh et al. Epidemiological Features of COVID-19 in Northwest Russia in 2021. *Viruses* 2022, *14*, 931

**DOI:** 10.3390/v15051190

**Published:** 2023-05-18

**Authors:** Anna Gladkikh, Vladimir Dedkov, Alena Sharova, Ekaterina Klyuchnikova, Valeriya Sbarzaglia, Olga Kanaeva, Tatyana Arbuzova, Nadezhda Tsyganova, Anna Popova, Edward Ramsay, Areg Totolian

**Affiliations:** 1 Saint Petersburg Pasteur Institute, 14 Ulitsa Mira, 197101 Saint Petersburg, Russia; angladkikh@gmail.com (A.G.); alenasharova21@gmail.com (A.S.); erithrophtalmis@gmail.com (E.K.); sbarzaglia.valeriya@gmail.com (V.S.); ol.kanaeva@gmail.com (O.K.); arbuzowa95@yandex.ru (T.A.); lukyanchuk_np@pasteurorg.ru (N.T.); warmsunnyday@mail.ru (E.R.); totolian@spbraaci.ru (A.T.); 2 Martsinovsky Institute of Medical Parasitology, Tropical and Vector Borne Diseases, Sechenov First Moscow State Medical University, 119435 Moscow, Russia; 3 Federal Service on Consumer Protection and Human Well-Being Surveillance, 127994 Moscow, Russia; depart@gsen.ru

## Text Correction

There was an error in the original publication [1]. In the first paragraph of the Results section, (per 100 K/day) should be (per 100 K/month). This error appears four times in this paragraph, and everywhere it should be (per 100 K/month).

A correction has been made to the Results Section, Paragraph 1:

In 2021, COVID-19 incidence in northwest Russia varied in waves, ranging from 258.8 (per 100 K/month) in April to 1185.8 in November. At the same time, increases in incidence with the achievement of local maxima were recorded in January (1113.5 per 100 K/month), June (822.3 per 100 K/month), and November (1185.8 per 100 K/month) (Figure 1).

The authors state that the scientific conclusions are unaffected. This correction was approved by the Academic Editor. The original publication has also been updated.
